# Tobacco Use, Risk Perceptions, and Characteristics of Adults Who Used a Heated Tobacco Product (IQOS) in the United States: Cross-Sectional Survey Study

**DOI:** 10.2196/57398

**Published:** 2025-02-07

**Authors:** Hui G Cheng, Brendan Noggle, Andrea R Vansickel, Edward G Largo, Pierpaolo Magnani

**Affiliations:** 1 Altria Client Services LLC Richmond, VA United States; 2 PMI R&D, Philip Morris Products S.A. Neuchâtel Switzerland

**Keywords:** United States Food and Drug Administration, FDA, IQOS, Tobacco Heating System, THS, heated tobacco products, modified risk tobacco products, MRTP, tobacco, nicotine, smokers, tobacco harm reduction, cross-sectional surveys, cigarettes

## Abstract

**Background:**

The Tobacco Heating System (THS; commercialized as IQOS) is a smoke-free heated tobacco product introduced in the United States in 2019 and authorized by the US Food and Drug Administration as a modified risk tobacco product (MRTP) in 2020. THS consists of a holder and specially designed tobacco sticks that are heated instead of burned to produce a nicotine-containing aerosol. THS was available in Atlanta, Georgia; Richmond, Virginia; Charlotte, North Carolina; the Northern Virginia region; and South Carolina before its market removal in November 2021.

**Objective:**

This study aims to describe selected sociodemographic characteristics and self-reported health history of adults who used IQOS (AUIs), their tobacco use patterns (eg, tobacco use history, exclusive and dual-use, and switching from cigarette smoking), their risk perceptions of the product, and their understanding of MRTP messages.

**Methods:**

The IQOS Cross-Sectional Postmarket Adult Consumer Study was a study of AUIs aged 21 years or older who were recruited from a consumer database via direct postal mail and emails. Participants completed the online survey between September and November 2021.

**Results:**

The survey was completed by 645 current and 43 former AUIs who had used at least 100 tobacco sticks (considered established THS use) before the assessment. Of the 688 participants, 424 (61.6%) were male, 502 (73.0%) were non–Hispanic White, and the mean age was 45 years. The vast majority (680/688, 98.8%) of AUIs had ever smoked combusted cigarettes before first trying THS and 628 (91.3%) had smoked cigarettes in the 30 days before first using THS. At the time of assessment, 161 (23.4%) reported using e-cigarettes (vs 229, 33.3%, before THS use), 92 (13.4%) reported smoking cigars (vs 114, 16.6%, before THS use), and 338 (49.1%) were still smoking after an average of 1 year of THS use. Among those currently using THS who were still smoking (n=298), 249 (83.6%) smoked fewer cigarettes compared with before first trying THS; 362 of 688 (52.6%) AUIs reported having no physical health conditions evaluated in this study and almost three-quarters reported having no mental health conditions. Among all AUIs, over 563 (81.8%) had never used a cessation treatment or had not used it in the past 12 months, and 555 (80.7%) AUIs demonstrated a correct understanding of the MRTP message and AUIs perceived THS as having a lower risk than cigarettes (43.8 vs 64.4 on a 100-point composite score scale).

**Conclusions:**

This study provides evidence that THS can help adult smokers in the United States completely switch away from cigarettes or reduce smoking.

## Introduction

Cigarette smoking is a leading contributor to mortality and morbidity worldwide [1]. In the United States, smoking contributes to an estimated 500,000 premature deaths and an expenditure exceeding US $170 billion in annual medical costs to treat smoking-related lung and cardiovascular illnesses [2]. Although quitting is the best way to mitigate health risks from smoking, it remains challenging for many smokers. Despite 70% of smokers wanting to quit smoking and more than half attempting each year [3], the majority of those who attempt to quit relapse within 12 months [4-7]. Individuals who smoke make many attempts before successfully quitting, rendering the quitting journey a protracted relapsing process [7,8]. Given that health risks from cigarette smoking are mainly attributed to harmful constituents generated through combustion, smokers who are unable or unwilling to quit may benefit from switching to smoke-free products such as heated tobacco products including the Tobacco Heating System (THS; commercialized as *IQOS* by Philip Morris Products S.A.) [9-12].

THS is a smoke-free tobacco product that heats specially designed tobacco sticks (commercialized as *HeatSticks* by Philip Morris Products S.A.) to temperatures below the level of combustion and produces an aerosol with substantially reduced (ie, 90%-95% lower, on average) levels of harmful and potentially harmful constituents compared with cigarette smoke while delivering similar levels of nicotine [13-15]. The product debuted in 2014 in Japan and was available in more than 80 countries as of September 2024.

Evidence collected to date supports the role of THS in tobacco harm reduction. The product was rapidly adopted by individuals who smoked in Japan [16], and recent data suggest that the majority of individuals who used THS have switched to exclusive use and stopped smoking [17]. Moreover, individuals who used heated tobacco products exclusively, as opposed to smoking, had more favorable health-related biomarker profiles compared with smokers [18]. In a prospective study of 38 Italian smokers with chronic obstructive pulmonary disease who started to use THS, 58% stopped smoking within 3 years, and those who were still smoking significantly reduced their cigarette consumption (mean cigarette consumption dropped from 21 per day at baseline to 3.7 per day at the last follow-up). Most importantly, consistent improvements were observed in respiratory symptoms, exercise tolerance, quality of life, and rate of disease exacerbations, whereas no such change was observed among individuals who smoked cigarettes during the same follow-up period [19]. These results suggest that switching from cigarette smoking to THS may be associated with long-term health benefits.

The US Food and Drug Administration (FDA) authorized the marketing of THS in the United States in April 2019. Subsequently, it granted a modified risk tobacco product (MRTP) order in July 2020 authorizing the use of the following reduced exposure claim:

“AVAILABLE EVIDENCE TO DATE:

- The IQOS system heats tobacco but does not burn it.
- This significantly reduces the production of harmful and potentially harmful chemicals.
- Scientific studies have shown that switching completely from conventional cigarettes to the IQOS system significantly reduces your body’s exposure to harmful or potentially harmful chemicals.”

THS was first introduced to the United States in Atlanta (Georgia) in October 2019. Marketing expanded to Richmond (Virginia) in January 2020, Charlotte (North Carolina) in July 2020, and northern Virginia and South Carolina as of May 2021 before it was removed from all US markets in November 2021 as a result of a Cease-and-Desist Order (CDO) issued by the United States International Trade Commission (ITC). (The ITC issued a CDO prohibiting the importation, marketing, sale, and distribution of *IQOS* devices and *HeatSticks* on September 29, 2021. To comply with the CDO, Philip Morris USA stopped marketing and selling the products as of November 28, 2021, in all channels.) Three types of tobacco sticks were sold: amber/regular (nonmenthol), green/smooth menthol (menthol), and blue/fresh menthol (menthol). At the end of August 2021, the tobacco sticks were available in 4842 third-party retail outlets, and the THS devices were available in 1235 third-party retail outlets.

This study aimed to (1) characterize adults who used *IQOS* (AUIs) and their history and patterns of tobacco use, as well as tobacco cessation treatment use; (2) describe risk perceptions of THS and the understanding of the MRTP claim; and (3) describe complete switching from cigarette smoking to THS, transitions to/back to cigarette smoking, and relevant quitting behaviors among AUIs. We used data from the 2021 *IQOS* Cross-sectional Postmarket Adult Consumer Study to provide the first evidence about AUIs, how AUIs used the product, and the potential role of such products in tobacco harm reduction in real-world settings in the United States.

## Methods

### Study Design, Study Population, and Recruitment

This was a cross-sectional study. The study population consisted of adults, aged 21 years or older, who had used at least 100 tobacco sticks with THS before the assessment. Individuals were excluded if (1) they were unable to read, speak, or understand English; (2) they or their first-degree relative were current or former employees of the tobacco industry, the Central Research Organization, or any market research firm; or (3) they or their first-degree relative were currently involved in litigation against any tobacco industry entity.

**Figure 1:**
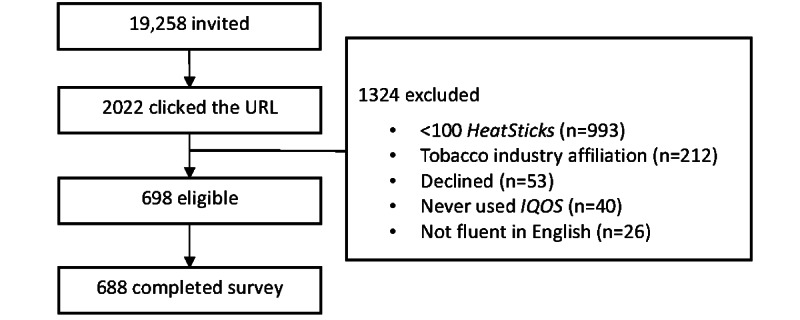
Flowchart depicting sample recruitment for the Cross-Sectional Postmarket Adult Consumer Study (2021). Participants sampled from the IQOS consumer database were primarily residing in Georgia, Virginia, North Carolina, or South Carolina, United States.

In this study, we recruited AUIs from a consumer database, which covered the majority of individuals who purchased an *IQOS* device. The *IQOS* Consumer Database is a database of registered *IQOS* consumers in the United States. These consumers entered the database by voluntarily registering their devices either at the point of sale or later by themselves. Based on the number of devices registered and sold, we estimated that approximately 70% of *IQOS* consumers were included in the *IQOS* Consumer Database. We sent invitations by both direct mail and email (if available) to all individuals in the database who agreed to be contacted for research purposes: 1 initial invitation and 1 reminder were sent to all potential participants. Figure 1 depicts the recruitment process and final sample. Data were collected between September 15 and November 14, 2021. In brief, of the 19,258 individuals invited, 2022 (10.50%) completed the screener; of the 698 eligible individuals who consented to participate, 688 (98.6%) completed the survey.

### Ethical Considerations

The study was reviewed (study protocol, questionnaire, and informed consent statement) and approved by the Sterling Institutional Review Board (IRB ID number: 9102-HCheng). This study was conducted in compliance with the study protocol and, where applicable, in accordance with the Insights Association Code of Standards and Ethics for Market Research and Data Analytics [20] and the International Chamber of Commerce/European Society for Opinion and Marketing Research’s (ESOMAR) International Code on Market, Opinion and Social Research [21]. All participants read the informed consent statement and voluntarily consented to participate in the study via electronic agreement. All data generated through this study were considered highly confidential by study staff and participants were identified only via unique participation identification numbers. Upon completion of the questionnaire, participants were provided instructions to obtain a US $40 electronic gift card.

### Assessment and Definitions

All eligible participants, regardless of the mode of recruitment, self-administered the questionnaire online. The assessment consisted of modules of questions on the history of use of various tobacco products (including THS, cigarettes, cigars, pipe, hookah, e-cigarettes, smokeless tobacco, and oral tobacco-derived nicotine products). Additional modules included risk perceptions and understanding of claim-related information, demographic characteristics, and diagnoses of selected physical and mental conditions.

In this study, we defined ever use as a “yes” response to the question “Have you EVER used/smoked...EVEN ONE TIME?” Current use was defined as “every day” or “some days” responses to the question “Do you now use/smoke...every day, some days, or not at all?” Former use was defined as a “yes” response to the ever-use question and a “not at all” response to the current-use question. Established cigarette smoking and THS use were defined as having smoked at least 100 cigarettes or used at least 100 tobacco sticks in a lifetime. Menthol/nonmenthol preference was assessed using the question “Which *one* type of *HeatSticks* are you *CURRENTLY* using *most often*?” Responses of “green menthol” (previously “smooth menthol”) and “blue menthol” (previously “fresh menthol”) were classified as preferring menthol; responses of “amber” (previously “regular”) were classified as preferring nonmenthol. We identified menthol/nonmenthol cigarette preference via questions about the type of cigarettes smoked most often.

In this study, we asked time-centered questions to establish a temporal sequence of smoking and THS use to assess changes in smoking behaviors after first trying THS. For example, we classified current smokers before first trying THS as individuals who answered “every day” or “some days” to the question “During the 30 days before you first tried *IQOS*, did you smoke cigarettes every day, some days, or not at all?”. Using this approach, we defined individuals who had *completely switched* as current established AUIs, who (1) had smoked at least 100 cigarettes in their lifetime, (2) were smoking every day or some days in the 30 days before first trying THS, (3) were not smoking at the time of assessment, and (4) had smoked their last cigarette after trying THS (via the question “Was the last time you smoked cigarettes...?” with response options “Before trying *IQOS* for the first time” and “After trying *IQOS* for the first time”).

Perceived health risks were assessed using the validated 18-item Perceived Health Risk scale, which has demonstrated good psychometric properties [22]. Two batteries of questions were asked for cigarette smoking and THS use, respectively. Each item was rated on a 5-point Likert-type scale ranging from 0 (no risk) to 4 (very high risk). An overall score was calculated by summing all items in the scale (possible range 0-72), with higher scores representing greater perceived health risk [22]. The understanding of the MRTP message was assessed using 2 questions shown in Textbox 1.

**Questions to assess understanding of the modified risk tobacco product message.**

- 1. Based on what you know or believe, please complete the following: Smokers who switch completely from cigarettes to IQOS:
  - Have more exposure to harmful or potentially harmful chemicals
  - Have the same exposure to harmful or potentially harmful chemicals
  - Have less exposure to harmful or potentially harmful chemicals (correct answer)
  - Have no exposure to harmful or potentially harmful chemicals
  - Don’t know

IF “HAVE LESS EXPOSURE...” SELECTED

- 2. Based on what you know or believe, what do smokers need to do in order to reduce their body’s exposure to harmful or potentially harmful chemicals?
  - Stop smoking cigarettes completely and only use IQOS (correct answer)
  - Smoke fewer cigarettes and also use IQOS
  - Keep smoking the same amount of cigarettes and also use IQOS
  - Don’t know

Demographic characteristics included sex (male or female), age, race/ethnicity, level of education, household income, employment status, and marital status. A lifetime history of physical conditions was assessed using the question “Have you ever been told by a doctor, nurse, or other health professional that you had...?” Options included cardiovascular conditions, respiratory conditions, cancers, and diabetes (see Table 2 for details). A lifetime history of mental conditions was assessed using 2 questions: “A mental illness or disorder refers to a wide range of mental health conditions or disorders that affect your mood, thinking, or behavior. Has a doctor, nurse, or other health professional ever told you that you had a mental health condition such as depression, stress, or problems with emotions?” and “Are you now taking medicine or receiving treatment from a doctor, nurse, or other health professional for a mental health condition or emotional problem?” All information was based on self-report.

### Analysis

Descriptive statistics were used to summarize outcome variables. For categorical variables, frequencies and proportions (95% CIs) were generated. For numeric variables (eg, age), means (95% CI) and medians (IQRs) were calculated. Estimates (eg, proportions and means) were presented for current and ever AUIs, respectively. Demographic characteristics and THS use behaviors were further stratified for AUIs who preferred menthol and nonmenthol tobacco sticks. Estimates are not shown for former established AUIs due to imprecision as a result of the small sample size (n=43). Missing values were kept as missing except for missing values from logic skips in which case, values were assigned based on the logic skip. For example, if a participant indicated that they had never used an ENDS (electronic nicotine delivery systems) product, then the current ENDS use question would be skipped and a “no” response value assigned. Data analyses were conducted using SAS version 9.4 (SAS Institute Inc.). The protocol for this study was published previously [23]. Data are available for interested researchers. Please contact the corresponding author for details.

## Results

### Characteristics of Study Participants

Of the 688 established AUIs, 273 (39.7%) resided in Georgia, 179 (26.0%) resided in North Carolina, 177 (25.7%) resided in Virginia, and 45 (6.5%) resided in South Carolina. Nominal numbers (n<5) of participants resided in other states. Of the total 688 participants, 424 (61.6%) were males, and the mean age was 45 years, with the majority between 35 and 54 years of age. On average, current established AUIs who preferred menthol tobacco sticks were younger than those who preferred nonmenthol tobacco sticks (mean age 43.1 years, 95% CI 42.0-44.3 vs 47.3 years, 95% CI 46.1-48.6). Of all established AUIs who had ever smoked cigarettes (n=680), 279 (41.0%; 95% CI 36.9%-44.3%) smoked menthol cigarettes most often. Individuals who were non–Hispanic White accounted for the majority of established AUIs (502/688, 73%). Differences in tobacco stick varieties were observed across race/ethnicity groups; greater proportions of non–Hispanic Whites preferred nonmenthol tobacco sticks, whereas greater proportions of non–Hispanic Black and non–Hispanic Asians preferred menthol varieties. Approximately two-thirds of established AUIs had an annual family income greater than US $60,000, 556 (80.8%) had at least some college education, and 547 (79.5%) were in the workforce. Table 1 provides more detailed information about the demographic characteristics of the sample.

**Table 1:** Demographic characteristics of established AUIs^a,b^.

Characteristics			Ever-established AUIs (n=688)		Current established AUIs (n=645)		Current established AUIs who prefer menthol tobacco sticks (n=337)		Current established AUIs who prefer nonmenthol tobacco sticks (n=8)
**Sex, n (%); 95% CI**									
	Male	424 (61.6); 58 to 65.3		394 (61.1); 57.3 to 64.9		205 (61.1); 55.9 to 66.3		188 (61.0); 55.6 to 66.5	
**Age (years)**									
	Mean (95% CI)	45.0 (44.1 to 45.9)		45.1 (44.3 to 46)		43.1 (42 to 44.3)		47.3 (46.1 to 48.6)	
	Median (IQR)	43.5 (36-54)		44 (36-54)		41 (35-50)		47 (39 to 55.5)	
	21-24, n (%); 95% CI	14 (2.0); 1.0 to 3.1		13 (2.0); 0.9 to 3.1		6 (1.8); 0.7 to 3.8		7 (2.3); 0.9 to 4.6	
	25-34, n (%); 95% CI	121 (17.6); 14.7 to 20.4		109 (16.9); 14 to 19.8		76 (22.6); 18.1 to 27		33 (10.7); 7.3 to 14.2	
	35-44, n (%); 95% CI	222 (32.3); 28.8 to 35.8		209 (32.4); 28.8 to 36		121 (35.9); 30.8 to 41		88 (28.6); 23.5 to 33.6	
	45-54, n (%); 95% CI	178 (25.9); 22.6 to 29.1		172 (26.7); 23.3 to 30.1		81 (24.0) 19.5 to 28.6		91 (29.6); 24.5 to 34.6	
	55-64, n (%); 95% CI	112 (16.3); 13.5 to 19		103 (16); 13.1 to 18.8		37 (10.9); 7.6 to 14.3		66 (21.4); 16.9 to 26	
	65+, n (%); 95% CI	41 (6.0); 4.2 to 7.7		39 (6.1); 4.2 to 7.9		16 (4.8); 2.5 to 7		23 (7.5); 4.5 to 10.4	
**Race/ethnicity, n (%); 95% CI**									
	Hispanic/Latino	40 (5.8); 4.1 to 7.6		39 (6.1); 4.2 to 7.9		20 (5.9); 3.4 to 8.5		19 (6.2); 3.5 to 8.9	
	Non–Hispanic White	502 (73.0); 70.0 to 76.3		463 (71.8); 68.3 to 75.3		212 (62.9); 57.8 to 68.1		251 (81.5); 77.2 to 85.8	
	Non–Hispanic Black	38 (5.5); 3.8 to 7.2		37 (5.7); 3.9 to 7.5		28 (8.3); 5.4 to 11.3		9 (2.9); 1.3 to 5.5	
	Non–Hispanic Asian	91 (13.2); 10.7 to 15.8		90 (13.9); 11.3 to 16.6		68 (20.2); 15.9 to 24.5		22 (7.1); 4.3 to 10.0	
	Non–Hispanic Native Hawaiian or other Pacific Islander	0 (0); <0.1 to 0.5		0 (0); <0.1 to 0.6		0 (0); <0.1 to 1.1		0 (0); <0.1 to 1.2	
	Non–Hispanic American Indian or Alaska Native	8 (1.2); 0.5 to 2.3		8 (1.2); 0.5 to 2.4		6 (1.8); 0.7 to 3.8		2 (0.7); 0.1 to 2.3	
	Non–Hispanic Other	9 (1.3); 0.6 to 2.5		8 (1.2); 0.5 to 2.4		3 (0.9); 0.2 to 2.6		5 (1.6); 0.5 to 3.8	
**Household income (US $), n (%); 95% CI**									
	<60,000	231 (33.6); 30.1 to 37.1		212 (32.9); 29.2 to 36.5		120 (35.6); 30.5 to 40.7		92 (29.9).8 to 35	
	60,000-74,999	67 (9.7); 7.5 to 12.0		63 (9.8); 7.5 to 12.1		35 (10.4); 7.1 to 13.6		28 (9.1); 5.9 to 12.3	
	75,000-99,999	124 (18); 15.2 to 20.9		118 (18.3); 15.3 to 21.3		59 (17.5); 13.5 to 21.6		59 (19.2); 14.8 to 23.6	
	100,000-149,999	125 (18.2); 15.3 to 21.1		117 (18.1); 15.2 to 21.1		65 (19.3); 15.1 to 23.5		52 (16.9); 12.7 to 21.1	
	≥150,000	95 (13.8); 11.2 to 16.4		90 (14); 11.3 to 16.6		35 (10.4); 7.1 to 13.6		55 (17.9); 13.6 to 22.1	
	Prefer not to answer	46 (6.7); 4.8 to 8.6		45 (7.0); 5.0 to 8.9		23 (6.8); 4.1 to 9.5		22 (7.1); 4.3 to 10.0	
**Education, n (%); 95% CI**									
	High school or less	132 (19.2); 16.2 to 22.1		125 (19.4); 16.3 to 22.4		62 (18.4); 14.3 to 22.5		63 (20.5); 16.0 to 25.0	
	Some college	196 (28.5); 25.1 to 31.9		175 (27.1); 23.7 to 30.6		89 (26.4); 21.7 to 31.1		86 (27.9); 22.9 to 32.9	
	Associate’s degree	85 (12.4); 9.9 to 14.8		82 (12.7); 10.1 to 15.3		51 (15.1); 11.3 to 19.0		31 (10.1); 6.7 to 13.4	
	Bachelor’s degree	179 (26.0); 22.7 to 29.3		171 (26.5); 23.1 to 29.9		93 (27.6); 22.8 to 32.4		78 (25.3); 20.5 to 30.2	
	Master’s degree	76 (11.1); 8.7 to 13.4		73 (11.3); 8.9 to 13.8		32 (9.5); 6.4 to 12.6		41 (13.3); 9.5 to 17.1	
	Professional school degree	4 (0.6); 0.2 to 1.5		4 (0.6); 0.2 to 1.6		1 (0.3); <0.1 to 1.6		3 (1); 0.2 to 2.8	
	Doctorate	9 (1.3); 0.6 to 2.5		9 (1.4); 0.6 to 2.6		4 (1.2); 0.3 to 3		5 (1.6); 0.5 to 3.8	
	Other	7 (1); 0.4 to 2.1		6 (0.9); 0.3 to 2.0		5 (1.5); 0.5 to 3.4		1 (0.3); <0.1 to 1.8	
**Employment status, n (%); 95% CI**									
	Employed for wages	439 (63.8); 60.2 to 67.4		412 (63.9); 60.2 to 67.6		223 (66.2); 61.1 to 71.2		189 (61.4); 55.9 to 66.8	
	Self-employed	108 (15.7); 13.0 to 18.4		104 (16.1); 13.3 to 19.0		47 (14.0); 10.3 to 17.7		57 (18.5); 14.2 to 22.8	
	Not employed	141 (20.5); 17.5 to 23.5		129 (20.0); 16.9 to 23.1		67 (19.9); 15.6 to 24.1		62 (20.1); 15.7 to 24.6	
**Marital status, n (%); 95% CI**									
	Married	370 (53.8); 50.1 to 57.5		355 (55.0); 51.2 to 58.9		179 (53.1); 47.8 to 58.4		176 (57.1); 51.6 to 62.7	
	Widowed	8 (1.2); 0.5 to 2.3		8 (1.2); 0.5 to 2.4		4 (1.2); 0.3 to 3.0		4 (1.3); 0.4 to 3.3	
	Divorced	100 (14.5); 11.9 to 17.2		93 (14.4); 11.7 to 17.1		42 (12.5); 8.9 to 16.0		51 (16.6); 12.4 to 20.7	
	Separated	15 (2.2); 1.1 to 3.3		12 (1.9); 0.8 to 2.9		8 (2.4); 1.0 to 4.6		4 (1.3); 0.4 to 3.3	
	Never married	116 (16.9); 14.1 to 19.7		109 (16.9); 14.0 to 19.8		68 (20.2); 15.9 to 24.5		41 (13.3); 9.5 to 17.1	
	Living with partner	60 (8.7); 6.6 to 10.8		50 (7.8); 5.7 to 9.8		27 (8.0); 5.1 to 10.9		23 (7.5); 4.5 to 10.4	
	Do not wish to answer	19 (2.8); 1.5 to 4.0		18 (2.8); 1.5 to 4.1		9 (2.7); 1.2 to 5.0		9 (2.9); 1.3 to 5.5	

^a^AUI: adult who used *IQOS.*

^b^Participants sampled from the *IQOS* consumer database were primarily residing in Georgia, Virginia, North Carolina, or South Carolina.

Just over half of AUIs reported they had never received any physical diagnoses included in this study (362/688, 52.6%; 95% CI 48.9%-56.4%; see Table 2 for more details). The most common conditions reported were hypertension (165/688, 24.0%; 95% CI 20.8%-27.2%) and high cholesterol (148/688, 21.5%; 95% CI 18.4%-24.6%). Approximately a quarter of participants reported they had ever had a mental health condition (181/688, 26.3%; 95% CI 23.0%-29.6%).

**Table 2:** Lifetime diagnosis of selected physical and mental health conditions among established AUIs^a,b^.

Lifetime diagnosis			Ever-established AUIs (n=688), n (%); 95% CI		Current established AUIs (n=645), n (%); 95% CI
**Physical condition**					
	A heart attack, also called myocardial infarction	10 (2.8); 1.5 to 4.0		17 (2.6); 1.4 to 3.9	
	Angina, also called angina pectoris (chest pain or discomfort)	12 (1.7); 0.8 to 2.7		11 (1.7); 0.7 to 2.7	
	Congestive heart failure	5 (0.7); 0.2 to 1.7		5 (0.8); 0.3 to 1.8	
	Coronary heart disease	7 (1.0); 0.4 to 2.1		7 (1.1); 0.4 to 2.2	
	High blood pressure (hypertension)	165 (24.0); 20.8 to 27.2		154 (23.9); 20.6 to 27.2	
	High cholesterol (hyperlipidemia)	148 (21.5); 18.4 to 24.6		139 (21.6); 18.4 to 24.7	
	Stroke	9 (1.3); 0.6 to 2.5		9 (1.4); 0.6 to 2.6	
	Any other heart condition or heart disease	7 (1.0); 0.4 to 2.1		7 (1.1); 0.4 to 2.2	
	Chronic obstructive pulmonary disease	28 (4.1); 2.6 to 5.6		28 (4.3); 2.8 to 5.9	
	Chronic bronchitis	25 (3.6); 2.2 to 5.0		23 (3.6); 2.13 to 5	
	Emphysema	5 (0.7); 0.2 to 1.7		4 (0.6); 0.2 to 1.6	
	Asthma	66 (9.6); 7.4 to 11.8		63 (9.8); 7.5 to 12.1	
	Apnea (pauses in breathing during sleep)	51 (7.4); 5.5 to 9.4		46 (7.1); 5.2 to 9.1	
	Any other respiratory or lung condition	2 (0.3); <0.1 to 1.1		2 (0.3); <0.1 to 1.1	
	Cancer	23 (3.3); 2.0 to 4.7		20 (3.1); 1.8 to 4.4	
	Diabetes	46 (6.7); 4.8 to 8.6		43 (6.7); 4.7 to 8.6	
	None of the above	362 (52.6); 48.9 to 56.4		342 (53.0); 49.2 to 56.9	
**Mental health condition**					
	Yes	181 (26.3); 23.0 to 29.6		166 (25.7); 22.4 to 29.1	
	No	477 (69.3); 65.9 to 72.8		450 (69.8); 66.2 to 73.3	
	Do not know	30 (4.4); 2.8 to 5.9		29 (4.5); 2.9 to 6.1	
**Taking medication or receiving treatment for a mental health condition or emotional problem**					
	Yes	121 (17.7); 14.9 to 20.6		113 (17.5); 14.6 to 20.5	
	No	548 (79.7); 76.6 to 82.7		516 (80.0); 76.9 to 83.1	
	Do not know	18 (2.6); 1.4 to 3.8		16 (2.5); 1.3 to 3.7	

^a^AUI: adult who used *IQOS*.

^b^Participants sampled from the *IQOS* consumer database were primarily residing in Georgia, Virginia, North Carolina, or South Carolina.

### Tobacco Use History and Cessation Treatment Use History

As shown in Tables 3 and 4, 680 of the 688 (98.8%) established AUIs had ever smoked cigarettes and 628 (91.3%) were currently smoking cigarettes before they tried THS for the first time. By contrast, at the time of assessment, only 338 (49.1%) were smoking. At the time of the assessment, 144 of 645 (22.3%) current established AUIs were also using e-cigarettes and 86 (13.3%) were also using cigars. Use of other tobacco products was relatively rare (≤5%, 33 or fewer/645). Current established smokers (n=326) had an average of 24.0 (95% CI 22.6-25.4) years of smoking history; former established smokers (ie, AUIs who had stopped smoking; n=329) had an average of 20.9 (95% CI 19.6-22.3) years of smoking history.

When asked about cigarette smoking during the 30 days before first trying THS, 519 (75.4%) participants indicated they were smoking every day, 109 (15.8%) were smoking some days but not every day, and 52 (7.6%) indicated they were not smoking cigarettes (including 16 participants who had not smoked cigarettes for at least 12 months, ie, former established smokers). Only a small proportion (n=16, 2.3%) of ever-established THS users were former established smokers before first trying THS. Of note, 11 of these 16 were using at least one other type of tobacco product during the 30 days before trying THS.

The vast majority of ever-established AUIs who were former established smokers at the time of the assessment became former smokers after first trying THS. Of the 329 former established smokers at the time of the assessment, 289 (87.8%) were smoking cigarettes during the 30 days before first trying THS or smoked after first trying THS, which suggested that they became former smokers after first trying THS. Of the 30 participants who did not smoke during the 30 days before first trying THS and indicated that the last time they smoked was before trying THS, 14 indicated they had not smoked for less than 1 month before trying THS, and 16 had not smoked for at least 12 months before first trying THS.

Approximately one-third of established AUIs were using e-cigarettes and about 114 (16.6%) were using cigars; use of other tobacco products was less common (ie, ≤44/688, <7%) before first trying THS.

With respect to cessation treatment, 330 out of 645 (51.2%; 95% CI 47.3%-55.0%) current established AUIs had never used any tobacco cessation treatment, 202 (31.3%; 95% CI 27.7%-34.9%) had used it more than 12 months ago, 39 (6.0%; 95% CI 4.2%-7.9%) used it within the past 12 months but more than 30 days ago, and 27 (4.2%; 95% CI 2.6%-5.7%) used it within the past 30 days at the time of the assessment.

**Table 3:** Types of tobacco products used by ever-established AUIs^a^ (n=688) before first trying the THS^b^ and at the time of assessment^c^.

Tobacco products	Tobacco use before first trying THS				Current tobacco use at the time of assessment
	Ever tried, n (%); 95% CI	Ever-established use, n (%); 95% CI	Current use, n (%); 95% CI	Current use, n (%); 95% CI	
Cigarettes	680 (98.8); 97.7-99.5	632 (91.9); 89.8-93.9	628 (91.3); 89.2-93.4	338 (49.1); 45.4-52.9	
Cigars	428 (62.2); 58.6-65.8	125 (18.2); 15.3-21.1	114 (16.6); 13.8-19.4	92 (13.4); 10.8-15.9	
Pipe filled with tobacco	151 (22.0); 18.9-25.0	32 (4.7); 3.1-6.2	18 (2.6); 1.4-3.8	17 (2.5); 1.3-3.6	
Hookah	225 (32.7); 29.2-36.2	29 (4.2); 2.7-5.7	44 (6.4); 4.6-8.2	34 (4.9); 3.3-6.6	
E-vapor products	493 (71.7); 68.3-75.0	298 (43.3); 39.6-47.0	229 (33.3); 29.8-36.8	161 (23.4); 20.2-26.6	
Smokeless tobacco	174 (25.3); 22.0-28.5	96 (14.0); 11.4-16.5	38 (5.5); 3.8-7.2	28 (4.1); 2.6-5.6	
Oral tobacco-derived nicotine products	143 (20.8); 17.8-23.8	36 (5.2); 3.6-6.9	41 (6.0); 4.2-7.7	27 (3.9); 2.5-5.4	
Any tobacco product other than THS	685 (99.6); 98.7-99.9	659 (95.8); 94.3-97.3	658 (95.6); 94.1-97.2	461 (67); 63.5-70.5	

^a^AUI: adult who used *IQOS.*

^b^THS: Tobacco Heating System.

^c^Participants sampled from the *IQOS* consumer database were primarily residing in Georgia, Virginia, North Carolina, or South Carolina.

**Table 4:** Types of tobacco products used by current established AUIs^a^ (n=645) before first trying the THS^b^ and at the time of assessment^c^.

Tobacco products	Tobacco use before first trying THS				Current tobacco use at the time of assessment
	Ever tried (n=645), n (%); 95% CI	Ever-established use (n=645), n (%); 95% CI	Current use (n=645), n (%); 95% CI	Current use (n=645), n (%); 95% CI	
Cigarettes	638 (98.9); 97.8 to 99.6	591 (91.6); 89.5 to 93.8	586 (90.9); 88.6 to 93.1	316 (49.0); 45.1 to 52.9	
Cigars	396 (61.4); 57.6 to 65.2	118 (18.3); 15.3 to 21.3	106 (16.4); 13.6 to 19.3	86 (13.3); 10.7 to 16.0	
Pipe filled with tobacco	138 (21.4); 18.2 to 24.6	30 (4.7); 3.0 to 6.3	17 (2.6); 1.4 to 3.9	17 (2.6); 1.4 to 3.9	
Hookah	209 (32.4); 28.8 to 36.0	27 (4.2); 2.6 to 5.7	41 (6.4); 4.5 to 8.2	33 (5.1); 3.4 to 6.8	
E-vapor products	462 (71.6); 68.2 to 75.1	275 (42.6); 38.8 to 46.5	211 (32.7); 29.1 to 36.3	144 (22.3); 19.1 to 25.5	
Smokeless tobacco	157 (24.3); 21 to 27.7	87 (13.5); 10.9 to 16.1	36 (5.6); 3.8 to 7.4	26 (4.0); 2.5 to 5.6	
Oral tobacco-derived nicotine products	128 (19.8); 16.8 to 22.9	32 (5.0); 3.3 to 6.6	35 (5.4); 3.7 to 7.2	23 (3.6); 2.1 to 5.0	
Any tobacco product other than THS	643 (99.7); 98.9 to >99.9	617 (95.7); 94.1 to 97.2	616 (95.5); 93.9 to 97.1	428 (66.4); 62.7 to 70.0	

^a^AUI: adult who used *IQOS*.

^b^THS: Tobacco Heating System.

^c^Participants sampled from the *IQOS* consumer database were primarily residing in Georgia, Virginia, North Carolina, or South Carolina.

### THS Use

#### Overall Use Behaviors

On average, AUIs had used THS for 1.2 years (mean 1.2, 95% CI 1.1-1.2 for all established AUIs; mean 1.2, 95% CI 1.1-1.3 for current AUIs). Among current AUIs (n=645), one-third were using THS only (n=217, 33.6%; 95% CI 30.0%-37.3%), 280 (43.4%; 95% CI 39.6%-47.2%) were using it and 1 other tobacco product (including 187, 29.0%, who were smoking cigarettes and 93, 14.4%, who were not smoking cigarettes), and 148 (23.0%; 95% CI 19.7%-26.2%) were using it and at least two other tobacco products (including 129, 20.0%, who were smoking cigarettes and 19, 2.9%, who were not smoking cigarettes). Of current AUIs, 443 (68.7%) used THS every day during the 30 days before the assessment. A greater proportion of current established THS users who had used the device for more than a year used THS daily compared with those who had used THS for a year or less (218/300, 72.7%, vs 225/345, 65.2%, *P*=.04). Current AUIs used a median of 15 tobacco sticks per day on days used (Table 5).

**Table 5:** Patterns of Tobacco Heating System use in the past 30 days at the time of the assessment^a^.

Patterns		Current established AUIs^b^ (n=645)	Current established AUIs who prefer menthol tobacco sticks (n=337)	Current established AUIs who prefer nonmenthol tobacco sticks (n=308)
**Number of days used, n (%); 95% CI**				
	0-9	36 (5.6); 3.8-7.4	14 (4.2); 2.0-6.3	22 (7.1); 4.3-10.0
	10-19	66 (10.2); 7.9-12.6	31 (9.2); 6.1-12.3	35 (11.4); 7.8-14.9
	20-29	100 (15.5); 12.7-18.3	52 (15.4); 11.6-19.3	48 (15.6); 11.5-19.6
	30	443 (68.7); 65.1-72.3	240 (71.2); 66.4-76.1	203 (65.9); 60.6-71.2
Number of days used, mean (95% CI)		25.9 (25.3-26.5)	26.3 (25.6-27.1)	25.4 (24.5-26.2)
Number of days used, median (IQR)		30 (25-30)	30 (26-30)	30 (22-30)
**Number of tobacco sticks used per day on days used, n (%); 95% CI**				
	<1	7 (1.1); 0.4-2.2	5 (1.5); 0.5-3.4	2 (0.7); 0.1-2.3
	1	4 (0.6); 0.2-1.6	2 (0.6); 0.1-2.1	2 (0.7); 0.1-2.3
	2	9 (1.4); 0.6-2.6	5 (1.5); 0.5-3.4	4 (1.3); 0.4-3.3
	3	17 (2.6); 1.4-3.9	9 (2.7); 1.2-5	8 (2.6); 1.1-5.1
	4	10 (1.55); 0.6-2.5	4 (1.2); 0.3-3	6 (2); 0.7-4.2
	5-9	104 (16.1); 13.3-19.0	55 (16.3); 12.4-20.3	49 (15.9); 11.8-20
	10-14	146 (22.6); 19.4-25.9	79 (23.4); 18.9-28	67 (21.8); 17.2-26.4
	15-19	94 (14.6); 11.9-17.3	49 (14.5); 10.8-18.3	45 (14.6); 10.7-18.6
	20+	250 (38.8); 35.0-42.5	126 (37.4); 32.2-42.6	124 (40.3); 34.8-45.7
Number of tobacco sticks per day on days used, median (IQR)		15 (10-20)	15 (10-20)	15 (10-20)
Number of tobacco sticks per day, median (IQR)		13 (7-20)	12.5 (8-20)	14 (7-20)

^a^Participants sampled from the *IQOS* consumer database were primarily residing in Georgia, Virginia, North Carolina, or South Carolina.

^b^AUI: adult who used *IQOS*.

#### Menthol and Nonmenthol Tobacco Stick Preferences

The majority of current AUIs had tried all 3 varieties of tobacco sticks (461/645, 71.5%; 437/645, 67.8%; and 359/645, 55.7%, for amber; green menthol; and blue menthol, respectively) by the time of the assessment. Amber was the most common variety tried first (344/645, 53.3%; 202/645, 31.3%; and 99/645, 15.3%, for green menthol and blue menthol, respectively); at the time of the assessment, nearly half indicated amber tobacco sticks as the variety currently used most often (308/645, 47.8%; 191/645, 29.6%; and 146/645, 22.6%, for green menthol and blue menthol, respectively).

When stratified by menthol and nonmenthol cigarette preference, we observed a clear preference for menthol tobacco sticks among participants who smoked menthol cigarettes most often. For example, among participants who formerly smoked menthol cigarettes most often (n=145), 135 (93.1%) used either green menthol or blue menthol tobacco sticks most often. Among participants who formerly smoked nonmenthol cigarettes most often (n=195), 144 (73.8%) used amber tobacco sticks most often. Similar patterns were observed among participants who currently smoked cigarettes: Among the 127 participants who currently smoked menthol cigarettes most often, 121 (95.3%) used menthol tobacco sticks most often; among the 204 participants who currently smoked nonmenthol cigarettes most often, 161 (78.9%) used nonmenthol tobacco sticks most often. There was little difference in the frequency and amount of tobacco stick use between those who preferred menthol and nonmenthol tobacco sticks (Table 5).

### Perceptions of THS

THS was perceived as having lower health risk as compared with cigarette smoking: the mean composite score of the Perceived Health Risk scale was 43.8 (95% CI 42.6-45.1) for THS and 64.4 (95% CI 63.0-65.7) for cigarette smoking.

With respect to understanding the MRTP message, among the 688 ever-established AUIs, 555 (80.7%; 95% CI 77.7%-83.6%) correctly identified “less exposure” to harmful or potentially harmful chemicals when switching completely from cigarettes to THS. Only 33 (4.8%; 95% CI 3.2%-6.4%) perceived “no exposure.” Other participants answered “the same exposure” (59/688, 8.6%; 95% CI 6.5%-10.7%), “more exposure” (6/688, 0.9%, 95% CI 0.3%-1.8%), or they “do not know” (35/688, 5.1%, 95% CI 3.5%-6.7%). Among those who correctly identified “less exposure” (n=555), 471 (84.9%; 95% CI 81.9%-87.9%) understood that “smokers must stop smoking completely and only use *IQOS*” to reduce their exposure to harmful or potentially harmful chemicals. Only 6 (1.1%; 95% CI 0.4%-2.3%) selected “keep smoking the same amount of cigarettes and also use *IQOS*.”

### Switching to THS and Transitions to/Back to Cigarette Smoking

#### Complete Switching

Among all current established AUIs (n=645), 191 (29.6%; 95% CI 26.1%-33.1%) met our predefined “complete switching” from cigarette smoking criteria, that is, participants who (1) had smoked at least 100 cigarettes in their lifetime, (2) were currently smoking during the 30 days before first trying THS, (3) were not smoking at the time of assessment, and (4) smoked their last cigarettes after trying THS. To note, this estimate is best interpreted as the proportion of current AUIs who had switched from cigarettes to THS from a cross-sectional assessment. It should not be interpreted as a “switching rate” as it does not provide any information about the “speed” of transition. Moreover, 350 of 688 (50.9%) AUIs were not smoking at the time of assessment (ie, 338/688, 49.1%, AUIs were also smoking; see Tables 3 and 4). The majority of individuals who were not smoking but did not meet the “complete switching” criteria were those who indicated that the last time they smoked cigarettes was within 30 days *before* first trying THS. (To meet the “complete switching” criteria, participants needed to indicate the last time they smoked was *after* first trying THS.) It is possible that these individuals immediately switched and did not smoke a cigarette once they started using THS. Therefore, we consider our estimate of “complete switching” to be conservative because it does not include those who stopped smoking immediately upon first trying the product.

When stratified by daily and nondaily smoking during the 30 days before first trying THS, we found similar proportions of AUIs who completely switched from cigarette smoking to THS between daily (ie, every day) and nondaily (ie, someday) smokers (158/519, 30.4%, and 33/109, 30.3%, respectively). Among all AUIs, we did not observe any differences in proportions who met the “complete switching” criteria between those who used menthol tobacco sticks and those who used nonmenthol tobacco sticks most often (102/358, 28.5%, and 89/330, 27.0%, respectively). Participants who completely switched from cigarettes to THS had used THS for a longer duration (mean duration 15.6 months) compared with those who did not meet the complete switching definition (mean duration 13.6 months; estimated difference 2.0, 95% CI 0.4-3.7, *P*=.01).

Among AUIs who were also smoking at the time of assessment (ie, dual-use; n=298), 249 (83.6%; 95% CI 79.4%-87.8%) indicated that they smoked fewer cigarettes per day at the time of assessment; 7 (2.3%; 95% CI 1.0%-4.8%) indicated they smoked more and 42 (14.1%; 95% CI 10.1%-18.0%) indicated they smoked the same number of cigarettes per day at the time of assessment compared with the 30 days before trying THS.

#### Transition to or Back to Cigarettes

In this study, we observed no relapse of cigarette smoking (defined as the current use of cigarettes and having smoked at least 100 cigarettes and having had not smoked cigarettes for <12 months before first trying THS); we observed 1 individual who reinitiated cigarette smoking (defined as the current use of cigarettes and having smoked at least 100 cigarettes and having had not smoked cigarettes for at least 12 months before first trying THS).

## Discussion

### Principal Findings

To our knowledge, this study is the first to describe the characteristics of AUIs and comprehensively assess use behaviors and risk perceptions relevant to THS use in a real-world setting among established AUIs in the United States. We found that AUIs tended to be male, non–Hispanic White or non–Hispanic Asian, and middle-aged (age 35-54 years), with at least some college education and a household income over US $60,000. More than half of AUIs reported no physical health conditions and the majority (548/688, 79.7%) reported not receiving treatment for any mental health conditions. Our results showed that the vast majority of established AUIs were existing smokers with an average of over 20 years of smoking history; 570 of 645 (88.4%) had either never used or had tried but stopped using tobacco cessation treatment. After an average of 1.2 years of THS use, almost half of AUIs were not smoking at the time of the assessment, and more than 80% (156/187, 83.4%) of participants who were still smoking indicated they had reduced their cigarette consumption. In addition, almost one-third met our strict definition for “complete switching” from cigarettes to THS and none reported relapse back to smoking. Finally, 555 of 688 (80.7%) participants perceived THS use as less harmful compared with cigarette smoking; few perceived it as risk-free and the vast majority understood the THS-modified risk claim. These findings provide firsthand empirical evidence in a real-world setting on THS consumers and their use behaviors during a relatively early stage of adoption in the United States.

### Interpretation of Findings

These findings are in line with evidence from other countries in that the vast majority of AUIs smoked cigarettes when they first tried THS, and few had never smoked a cigarette [24,25]. Our findings suggested that THS can facilitate adults who smoked cigarettes—especially those who did not use cessation treatment or did not successfully quit smoking after trying cessation treatment—to switch to THS.

Previous studies have found that young adults were more likely to use e-vapor products compared with older adults [26,27]. By contrast, we found that those who used THS tended to be middle-aged or older adults with a relatively long smoking history. In addition, the majority of current established AUIs had never tried a cessation treatment and another 202 of 645 (31.3%) tried but discontinued cessation treatment more than 12 months ago. Therefore, THS may be particularly beneficial to those who are older, have a long history of smoking, and did not use or were not successful in using tobacco cessation treatment. Along with the finding of minimal uptake among individuals who had never smoked cigarettes, our results support the FDA’s conclusion that marketing this product is appropriate for the protection of public health.

With respect to product use, the majority of AUIs used it daily and used more than 15 tobacco sticks per day on days used. This level of consumption was generally in line with cigarette consumption among smokers in national surveys [28-30], and daily use of THS has been associated with switching [31,32]. Taken together, these results suggest the replacement of cigarettes with THS use.

The correct perception of risk can play an integral role in switching behaviors. In line with the large body of literature showing that individuals who perceive smoke-free tobacco products as less harmful compared with cigarettes are more likely to switch to such products from cigarette smoking [33,34], a previous study documented that individuals who used THS exclusively (rather than smoking) were more likely to perceive it as less harmful than cigarettes, compared with those who used THS and smoked cigarettes (ie, dual-use) [31]. Given this context, it is encouraging to observe that over 80% (555/688, 80.7%) of participants in this study correctly identified the reduced exposure to harmful or potentially harmful chemicals when switching completely from cigarettes to THS, and few participants incorrectly identified “no exposure.”

With respect to tobacco stick varieties, we found an interesting pattern suggesting that menthol tobacco sticks were used and preferred not only by individuals who preferred menthol cigarettes, but also by a sizable portion of those who preferred nonmenthol cigarettes. That is, although amber (or original) was the most commonly ever used (456/645, 70.7%) and first tried (342/645, 53.0%) variety, the 2 menthol varieties accounted for the majority of varieties used most often at the time of assessment. This aligns with the observation that approximately 1 in 5 individuals who preferred nonmenthol cigarettes preferred menthol tobacco sticks; by contrast, only 1 in 20 individuals who preferred menthol cigarettes preferred amber (or original). Although it is not clear why menthol tobacco sticks were more commonly preferred than amber, such findings support the potential role of menthol varieties in continued use. In this study, we found that ever and current use of other tobacco products was more common among participants of this study (with e-vapor products being the most common) compared with that among smokers based on estimates from national surveys [35]. This may suggest that individuals open to trying and using other tobacco products were more likely to try and use THS. When compared with the time before trying THS, we observed a ~30% (229/688 vs 161/688) reduction in e-cigarette use. It is possible that some participants were using e-vapor products to help stop cigarette smoking but were not successful; after initiating THS use, they stopped using e-vapor products. Future studies that assess reasons to stop using other tobacco products will shed new light on this topic.

### Limitations

Our findings should be interpreted with the following limitations in mind. First, the study is based on self-reported information. Although we are not aware of any validation studies on self-reported use behaviors, the larger literature supports the reasonable validity of self-reported behaviors in tobacco research [36]. In addition, many measures relied on recalled information, so recall bias cannot be ruled out. We tried to minimize recall bias by (1) asking about prominent behaviors generally easy to recall, such as whether they smoked cigarettes during the 30 days before first trying THS, and (2) limiting the length of the recall period (eg, the past 30 days). Second, it is estimated that approximately 70% of individuals who purchased THS registered themselves in the database. Moreover, of all individuals (N=19,258) invited to complete the eligibility assessment, only 2022 (10.50%) did so. It is unknown whether individuals who registered in the database or responded to the invitation are representative of all AUIs in the United States. Also, due to limited distribution, it is not clear whether these results would apply to all adults who smoke in the United States. Nonetheless, we provided the first estimates of perceptions and behaviors related to THS use from a sample large enough to produce meaningful results. Third, the study was conducted among AUIs, so we cannot provide information about the prevalence of THS use in the general US population. Fourth, no definitive evidence can be drawn for causal relationships from this cross-sectional study. Perhaps future longitudinal studies can explore changes in behavior over time among individuals who adopt THS. Finally, it is noteworthy that during the recruitment period, a notification was sent to consumers on October 13, 2021, informing them that the product would become unavailable after November 28, 2021. In consideration of the potential impact of this communication on consumer behaviors, we also conducted analyses only using data collected before that date (n=463). Results from this subsample were generally in line with the results for the full sample.

### Conclusions

Our results provide supportive evidence for the potential of THS to help individuals switch from smoking by comprehensively assessing use behaviors and risk perceptions in a real-world setting among established AUIs in the United States.

## Abbreviations

AUI: adult who used IQOS
CASRO: Council of American Survey Research Organization
CDO: Cease-and-Desist Order
ENDS: electronic nicotine delivery system
ESOMAR: European Society for Opinion and Marketing Research
FDA: Food and Drug Administration
ITC: United States International Trade Commission
MRTP: modified risk tobacco product

## Availability of Data and Materials

The datasets used or analyzed during this study are available from the corresponding author upon reasonable request.
